# Supplementary material for: Separation of Five Flavonoids from Aerial Parts of Salvia Miltiorrhiza Bunge Using HSCCC and Their Antioxidant Activities
Source: Molecules. 2019 Sep 23;24(19):3448. doi: 10.3390/molecules24193448 (PMC6804221; doi:10.3390/molecules24193448)
Supplement: Supplementary file 1 [file molecules-24-03448-s001.pdf]

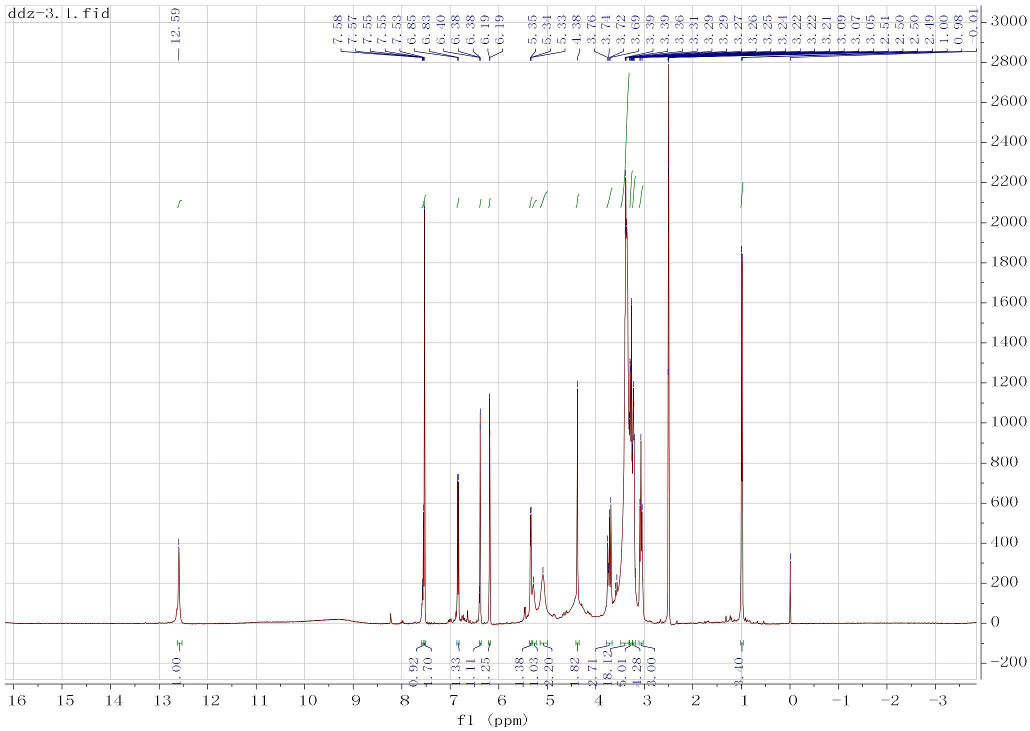

Figure S1. ^{1}H-NMR spectra of compound 1

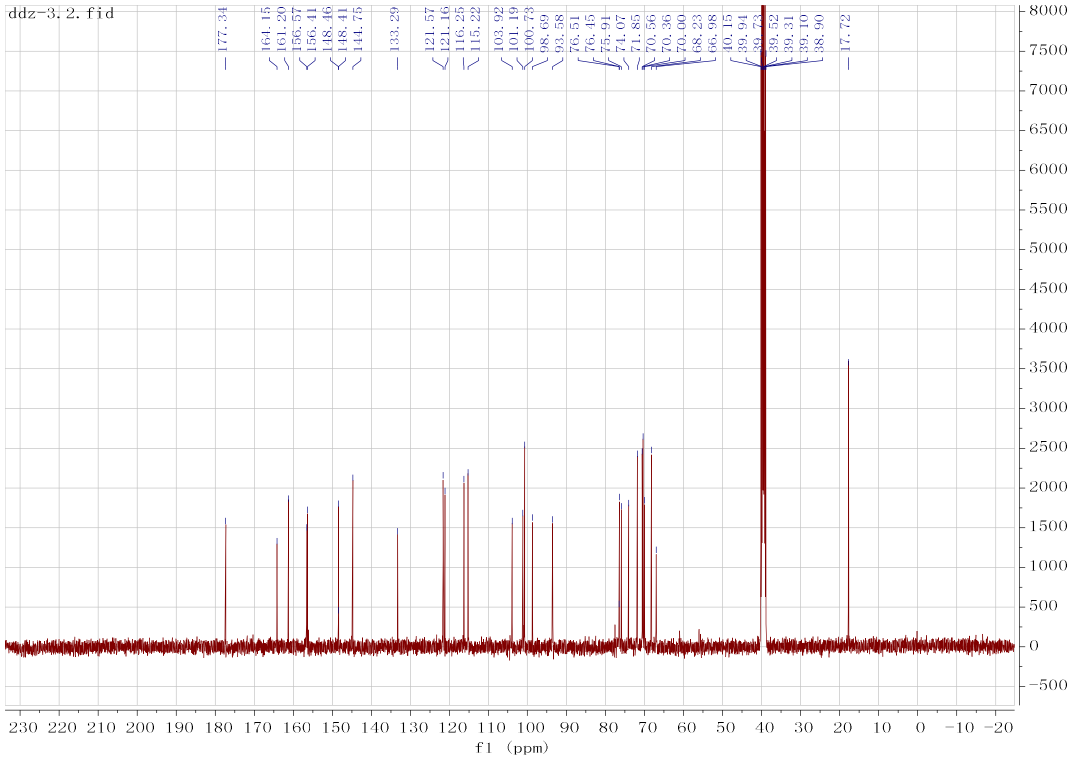

Figure S2. ^{13}C-NMR spectra of compound 1

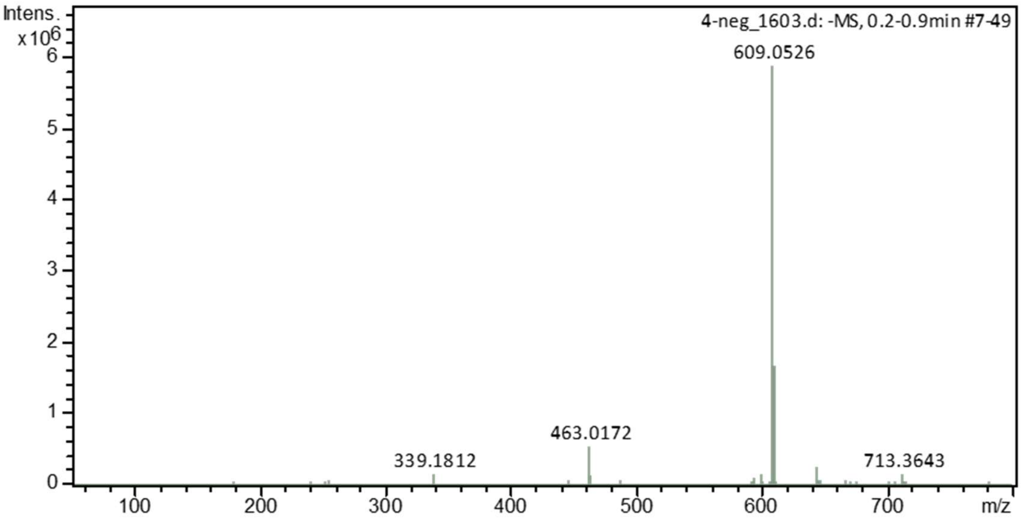

Figure S3. ESI-MS of Compound 1

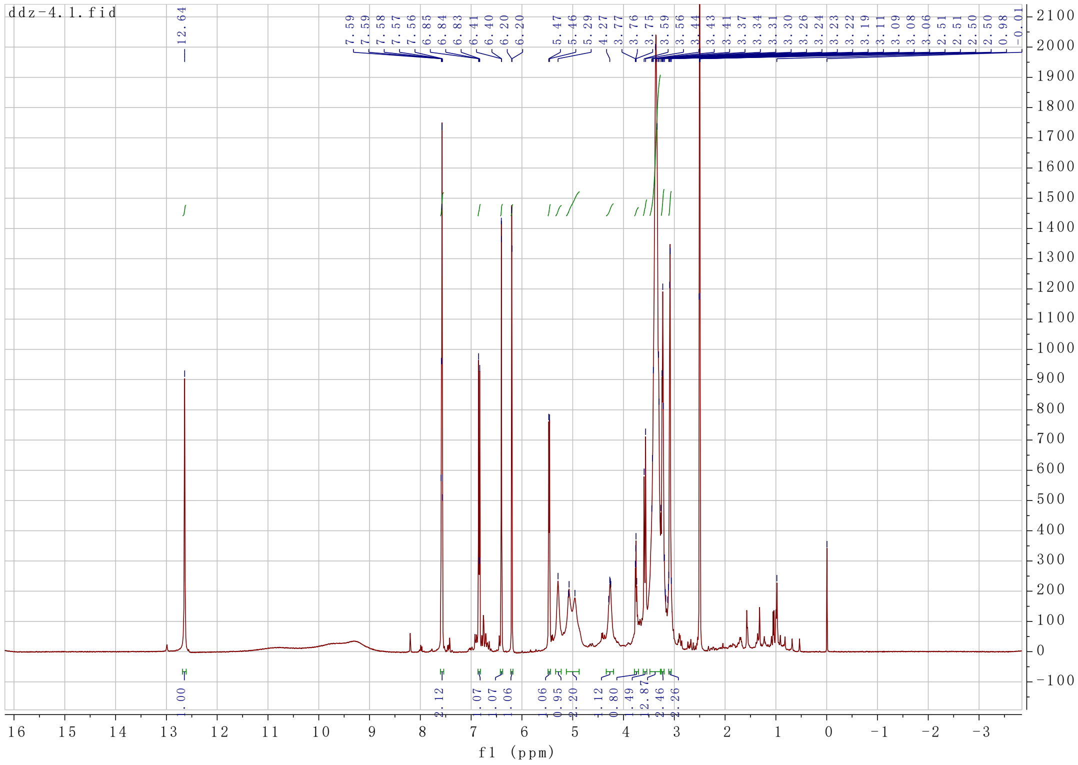

Figure S4. ^{1}H-NMR spectra of compound 2

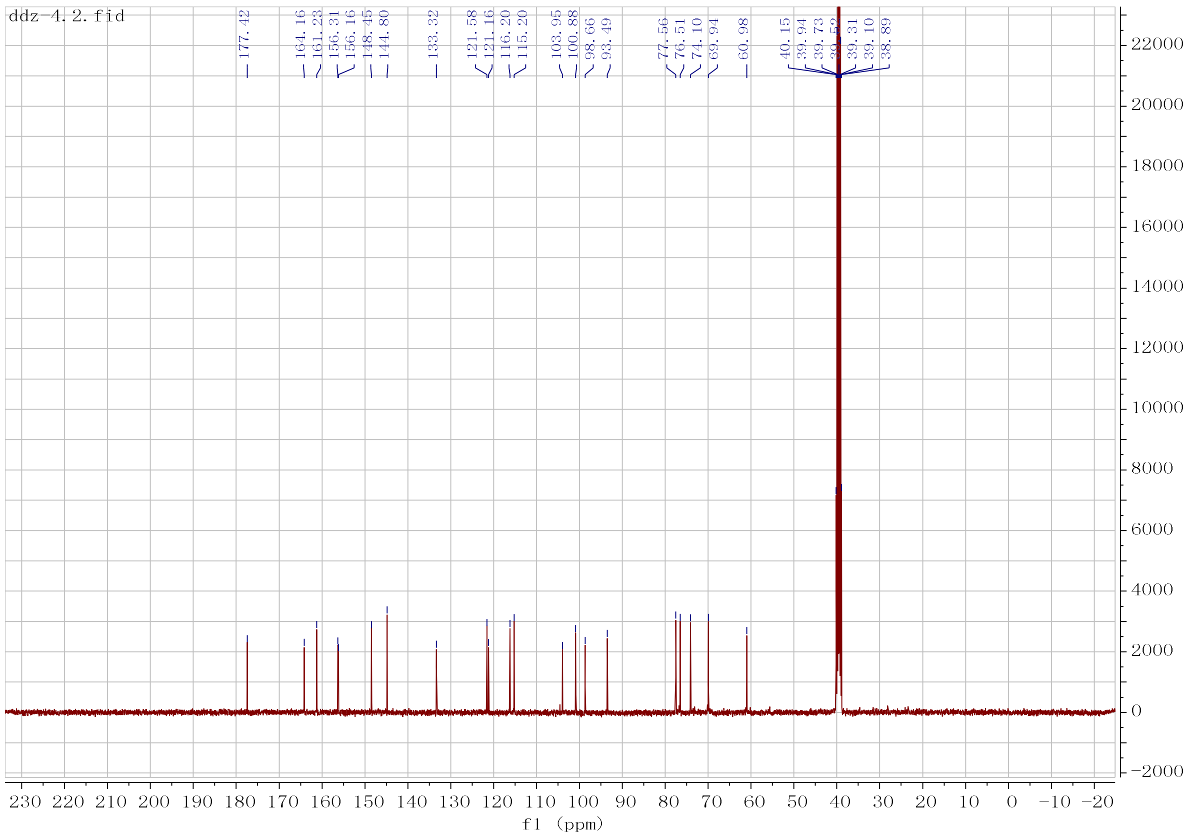

Figure S5. ^{13}C-NMR spectra of compound 2

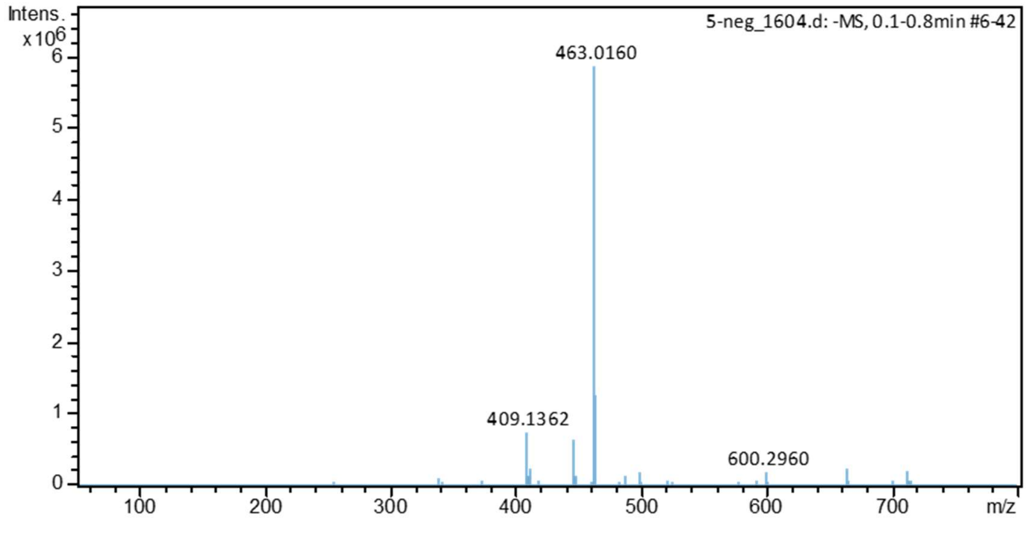

Figure S6. ESI-MS of Compound 2

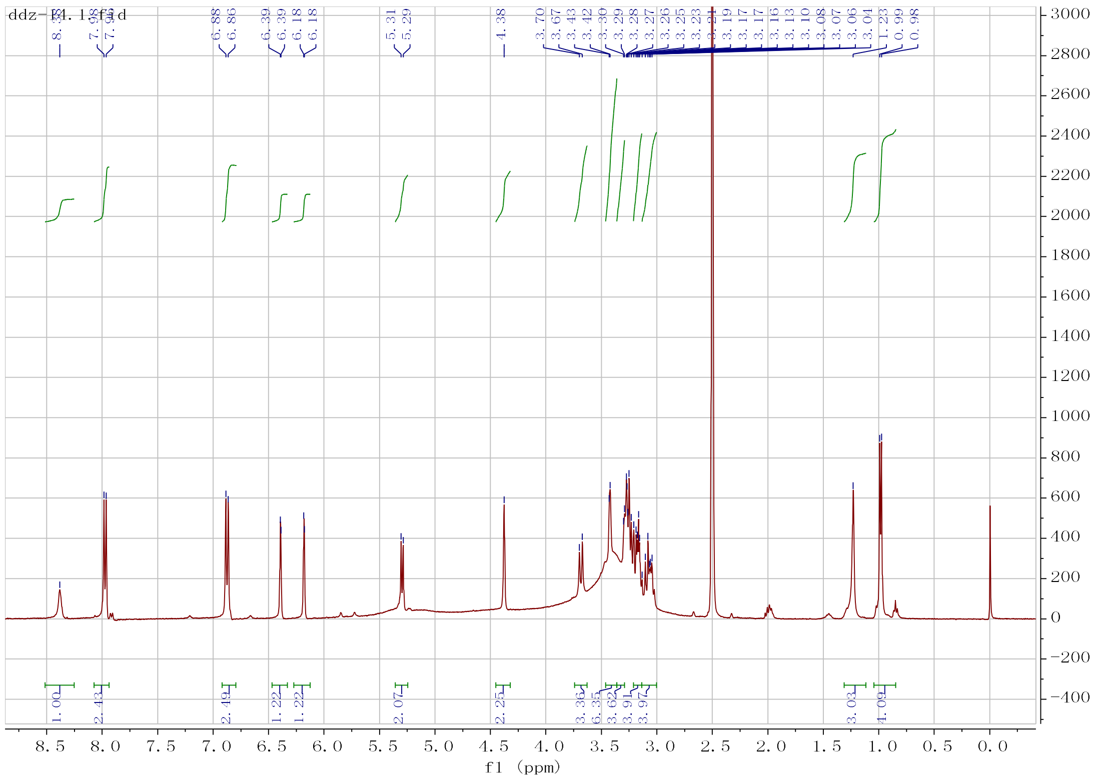

Figure S7. ^{1}H-NMR spectra of compound 3

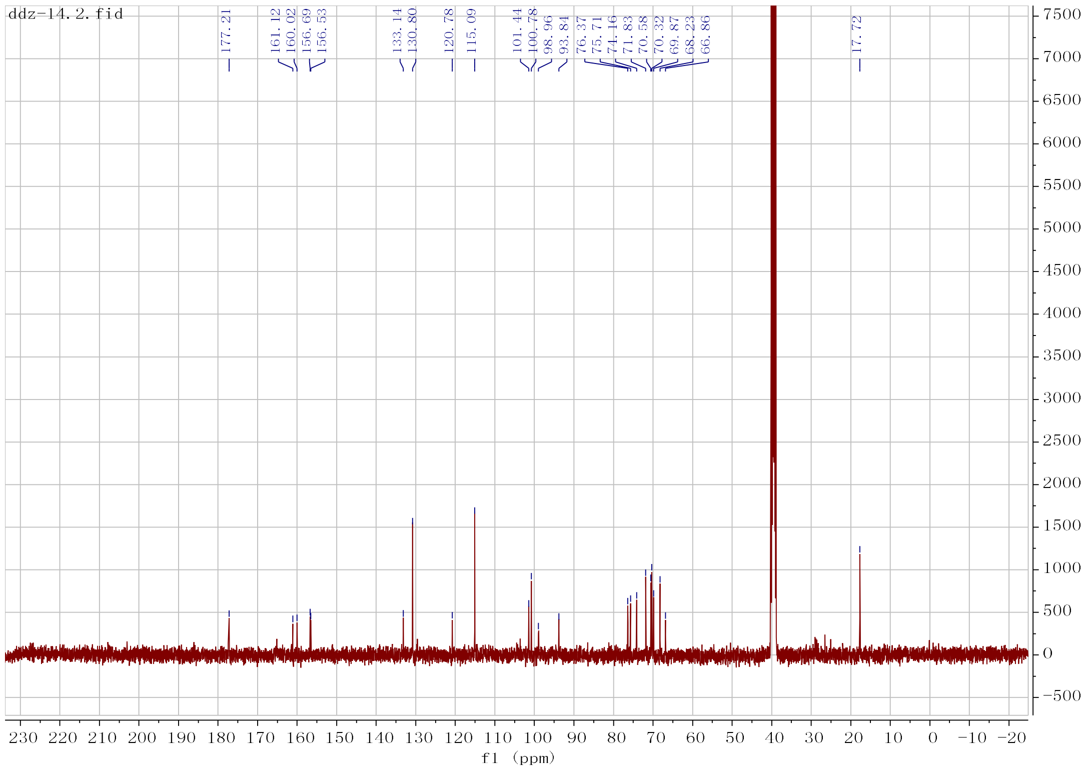

Figure S8. ^{13}C-NMR spectra of compound 3

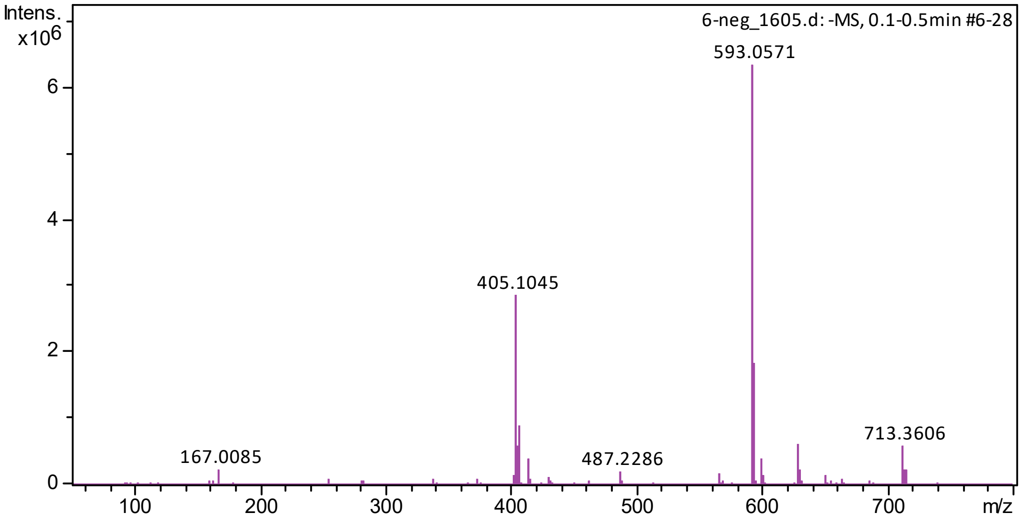

Figure S9. ESI-MS of Compound 3

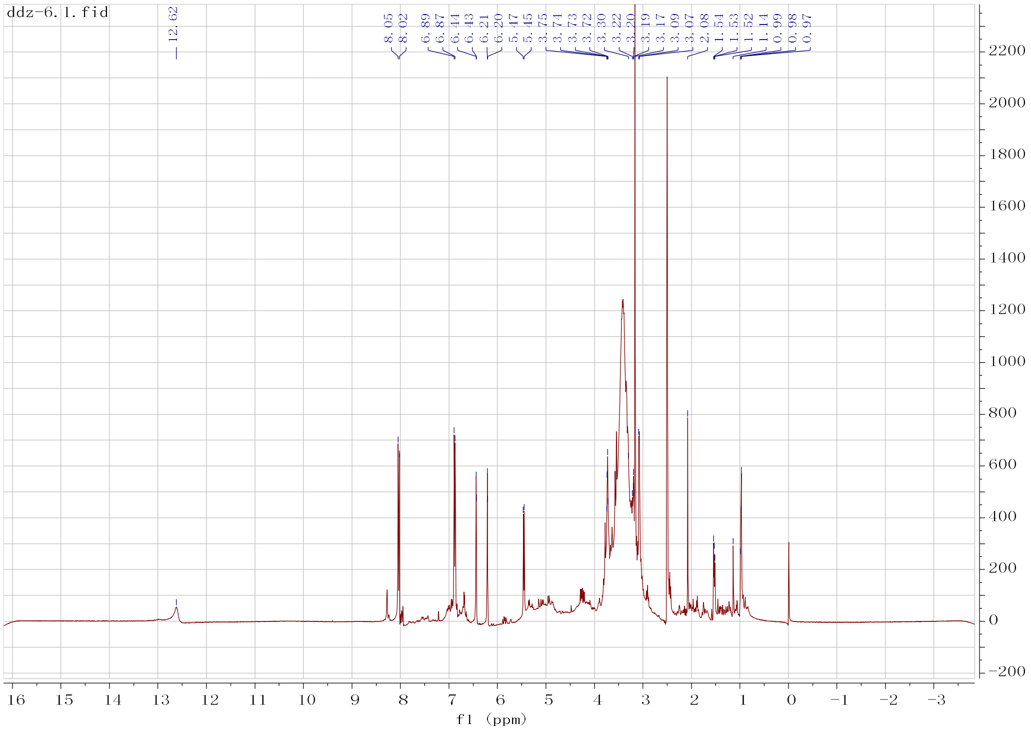

Figure S10. ^{1}H-NMR spectra of compound 4

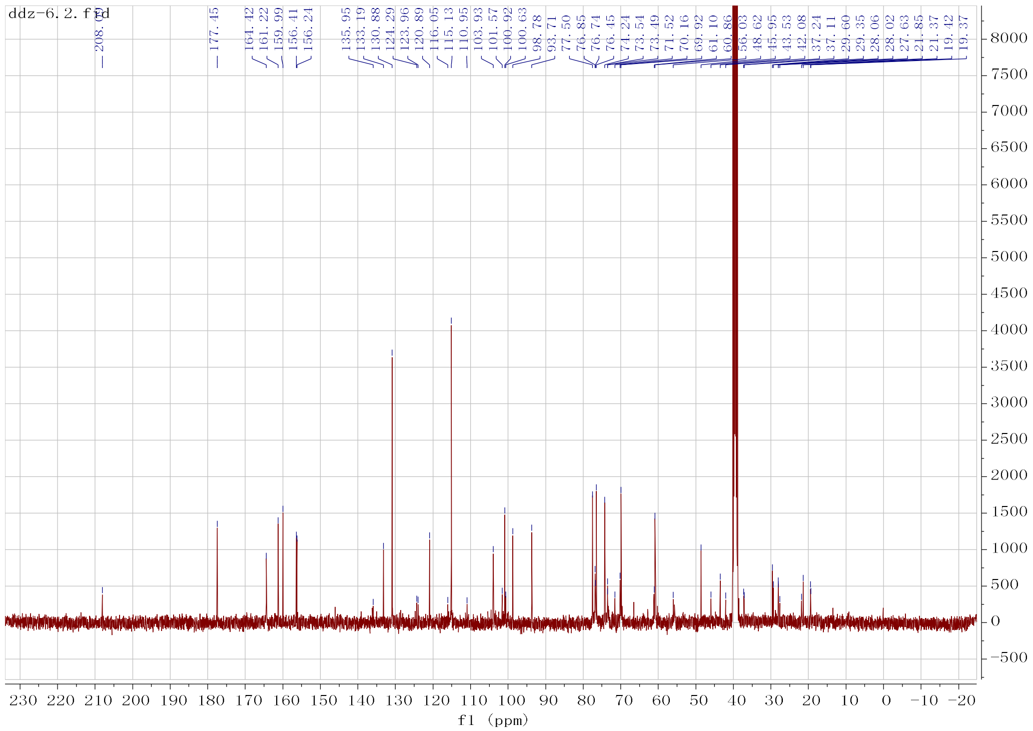

Figure S11. ^{13}C-NMR spectra of compound 4

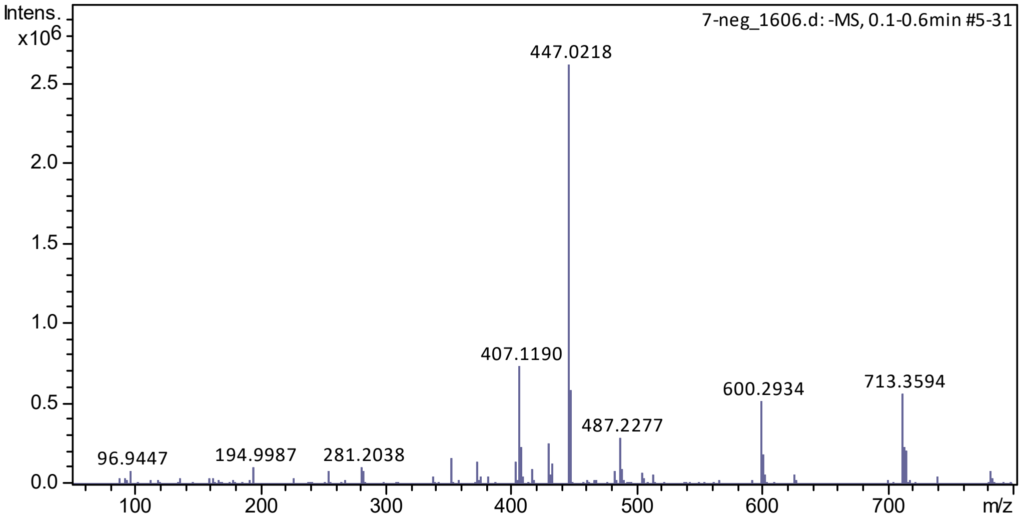

Figure S12. ESI-MS of Compound 4

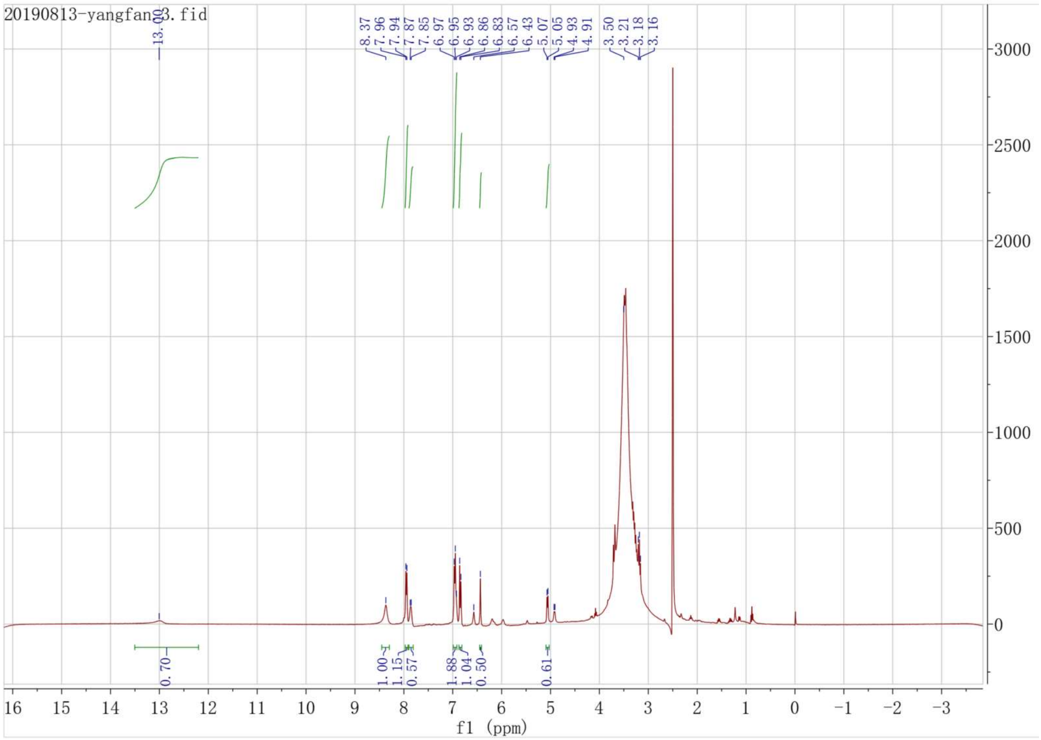

Figure S13. ^{1}H-NMR spectra of compound 5

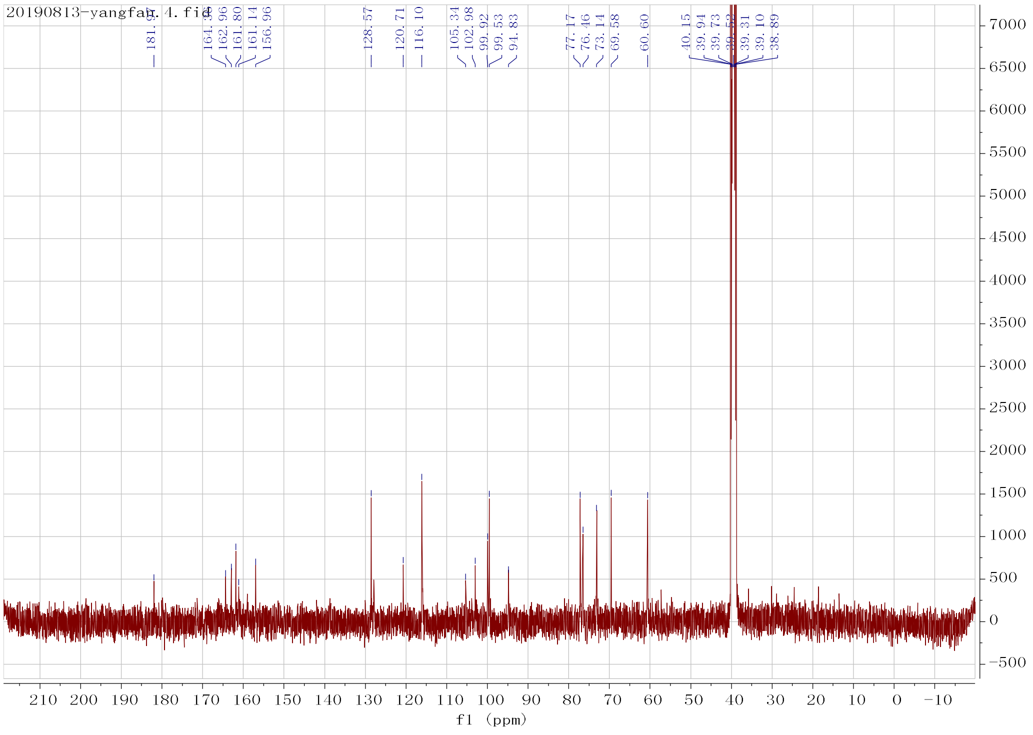

Figure S14. ^{13}C-NMR spectra of compound 5

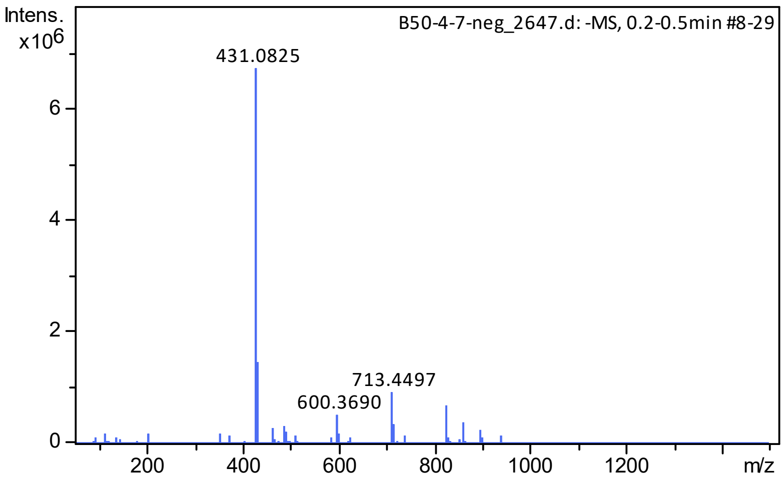

Figure S15. ESI-MS of Compound 5
